# Molecular Imprinting Polymer-Based Sensing of Neonicotinoids

**DOI:** 10.3390/s25237251

**Published:** 2025-11-27

**Authors:** Jelena Golijanin, Diane Hyewoo Lee, Riley Y. Li, Soha Ahmadi

**Affiliations:** 1 Department of Chemistry, University of Toronto, Toronto, ON M5S 3H6, Canada; jelena.golijanin@mail.utoronto.ca (J.G.); hyewoo.lee@mail.utoronto.ca (D.H.L.); riley.li@mail.utoronto.ca (R.Y.L.); 2 Department of Chemical Engineering & Applied Chemistry, University of Toronto, Toronto, ON M5S 3E5, Canada

**Keywords:** molecularly imprinted polymers, neonicotinoids, electrochemical sensors, optical sensors, voltammetry, potentiometry, photoelectrochemical sensing, fluorescence sensing, surface-enhanced Raman scattering

## Abstract

Neonicotinoids are a novel class of insecticides that exhibit environmental persistence and off-target effects on both humans and ecosystems. Therefore, there is a need for sensitive and selective sensors to monitor concentrations of neonicotinoids in environmental water and soil systems. Molecularly imprinted polymer (MIP)-based sensors are an emerging technology with strong potential for reliable, sensitive, and selective detection of neonicotinoids. Moreover, MIPs are versatile and compatible with a wide range of analytical techniques, which can further enhance measurement capabilities in the development of practical and robust sensors. Despite this promise, many routes remain underexplored for neonicotinoid detection. This review reports on the current state of neonicotinoid chemical sensors and detection methods using MIPs and highlights potential applications of MIP-based approaches as cost-effective and easy-to-operate solutions for monitoring neonicotinoids. Recent sensors incorporating MIPs and electrochemical or optical techniques for neonicotinoid detection are described and compared. Approaches employing magnetic solid-phase extraction and quartz crystal microbalance are also discussed. Additionally, the influence of monomer choice for MIP synthesis, as well as the use of additives and nanomaterials for sensor construction and analyte detection, is reviewed. These methods may promote sustainability, reusability, ratiometric or simultaneous detection of neonicotinoids, and sensor portability for on-site monitoring.

## 1. Introduction

### 1.1. Neonicotinoids

**Figure 1:**
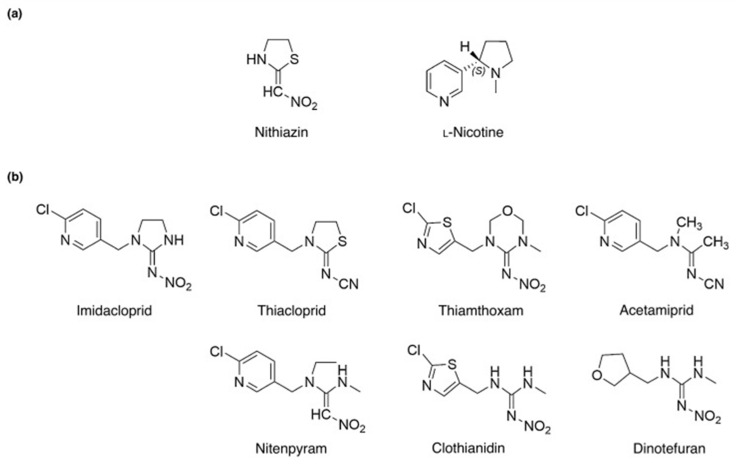
Chemical structure of neonicotinoids and their related compounds. (**a**) Chemical structure of nithiazine and nicotine. (**b**) Chemical structure of various neonicotinoids. Reproduced with permission from Ihara et al. (2018) [1] © 2018 Elsevier Inc.

Neonicotinoids are an emerging class of insecticides that have been commercialized since the early 1900s [1]. They are considered emerging insecticides despite being invented a century ago due to their recent uptick in use, accounting for 24% of the global insecticide market share in 2018 [2]. The increased use and importance of neonicotinoids are primarily due to the development of resistance to other insecticides, such as organophosphorus and carbamates, resulting from long-term use [3]. Neonicotinoids can overcome this resistance by acting through different insecticidal mechanisms; specifically, they target insect postsynaptic nicotinic acetylcholine receptors to hinder nerve conduction, rather than inhibiting acetylcholinesterase activity as organophosphorus and carbamate insecticides do [2]. The structures of common neonicotinoids and their precursors (nithiazine, nicotine) are shown in Figure 1.

However, the widespread use of neonicotinoids has led to their increased prevalence in food and environmental samples, including surface water, groundwater, and soil [4,5]. Recent studies have also shown that neonicotinoids are harmful to pollinators such as honeybees and bumble bees and to humans [6]. These studies have shown health impacts on pollinators’ reproduction, development, metabolism, and nervous system [1,4,5]. Consequently, with growing evidence of their potential harm and with increasing regulation from governments, the development of cost-effective, sensitive, point-of-care neonicotinoid sensors is critical to support regulatory efforts and protect both human and ecosystem health.

### 1.2. Molecularly Imprinted Polymers (MIPs)

Molecularly imprinted polymers (MIPs), more broadly known as molecular imprinting technology (MIT), were first developed by Wulff and Sarhan in 1972 [7]. MIT is based on the design of synthetic materials that contain recognition sites artificially designed for the selective rebinding of a target molecule. MIPs provide selective binding functionality via a polymeric matrix. Current applications of MIT have been dominated by polymer materials, as MIPs have significant advantages for detecting organic compounds with good selectivity, strong physical robustness, low cost, good thermal stability, and easy preparation [8].

While the most well-studied applications of MIPs have been in ample preparation, small molecule separation, and drug delivery, recent research has highlighted their potential as recognition elements for small molecules such as drugs and pesticides. This is due to their high specificity and sensitivity, which enable reliable trace-level detection in complex sample matrices without extensive pretreatment [9,10]. Incorporating MIPs into biosensors and chemical sensors would open new possibilities for in situ contaminant monitoring and rapid point-of-care analysis.

**Figure 2:**
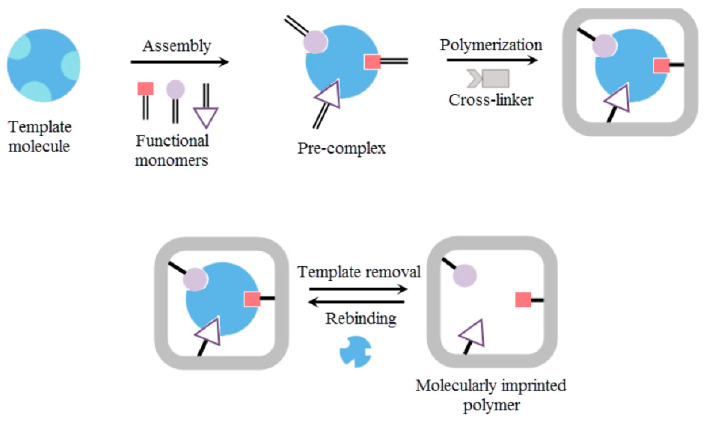
Overview of the process of developing MIPs. Developing MIPs requires a feasible polymerization protocol to imprint the target analyte, along with strong template-monomer interactions. Reproduced from Saylan et al. (2019) [11], under the terms of the Creative Commons Attribution (CC BY) license.

The synthesis of MIPs involves the polymerization of a functional monomer in the presence of crosslinkers and, most importantly, a template molecule. During polymerization, the template molecule can be covalently or non-covalently bonded to the functional monomer. Polymerization can occur through various mechanisms, such as electropolymerization, bulk, emulsion, suspension, and precipitation polymerization [9,10]. After polymerization, the template molecules are removed, resulting in the formation of microcavities complementary in shape, size, and spatial orientation of the template molecules [10]. The choice of monomer depends on both the chemical structure of the template molecule and the medium in which the MIP is intended to function (aqueous, organic). Strong template-monomer interactions are desired, as they enhance the affinity between the analyte and the monomer, thereby improving sensitivity in sensing applications [10]. The general process of developing MIPs is shown in Figure 2.

In designing MIP-based sensors, not only are the MIP systems important, but the signal enhancement and detection methods are also critical in developing portable, cost-effective, sensitive sensors. Some signal enhancement strategies include the use of nanoparticles, quantum dots, or other nanomaterials to amplify the signal in response to MIP binding with the target analyte [9]. The choice of enhancement strategy is closely linked to the detection method, which may include electrochemical methods such as voltammetry, amperometry, and electrochemical impedance spectroscopy, as well as optical methods such as fluorescence, colorimetry, and chemiluminescence [8,12].

In recent years, numerous new MIP-based sensors have been developed for the detection of neonicotinoids in food and environmental samples. Given the growing concerns about neonicotinoid contamination and its impact on both the ecosystem and human health, there is an urgent need for reliable detection tools. This review aims to provide an overview of the current state of research in this emerging field, with particular emphasis on the design of highly specific and sensitive MIP-based chemical sensors for neonicotinoid detection.

## 2. Electrochemical Sensors

Electrochemical sensing methods for neonicotinoid detection using MIPs have gained attention due to rapid responses, enabling real-time monitoring, and their potential for instrument miniaturization, which can reduce costs. Typical signal production strategies include direct detection via reduction of the neonicotinoid nitro group or binding of the analyte to electroactive materials [3].

While most MIP-based electrochemical sensors for neonicotinoid detection have relied on voltammetric techniques, several potentiometric, photoelectrochemical and impedance-based sensors have also been reported. Recent research has focused on improving sensor sensitivity and selectivity through MIP-based working electrode modifications, although efforts have also been made to utilize more environmentally friendly materials, reduce sample preparation, and improve sensor reusability.

### 2.1. Voltammetric Sensors

Voltammetry-based sensors operate by measuring the current response while sweeping the potential applied to the working electrode [13]. Voltammetry-based sensors are developed by modifying the working electrode to provide a selective recognition surface for detecting the target analyte. Common working electrodes include gold, glassy carbon, and graphite, which provide suitable surfaces for modification. Several voltammetry techniques are employed in sensor development, with cyclic voltammetry (CV), differential pulse voltammetry (DPV), and square wave voltammetry (SWV) being the most widely used [14]. While CV is often applied to monitor sensor fabrication and surface modification, pulse voltammetry techniques such as DPV and SWV are typically preferred for analyte detection due to their higher sensitivity [14,15]. The development of voltammetry-based MIP sensors largely relies on effective electrode surface modification, as the selectivity and sensitivity of the sensor are strongly influenced by both the design of the modification strategy and the MIP formulation. These sensors are particularly attractive for detecting neonicotinoids because they offer rapid responses, high sensitivity, and the potential for miniaturization and cost-effective instrumentation. However, challenges remain, including interference from complex sample matrices and the need for careful optimization of electrode surfaces to ensure reproducibility and long-term stability [14].

In 2024, Mutlu et al. developed a sensitive and selective DPV sensor for acetamiprid (ACT) detection in several fruits, employing an electropolymerized pyrrole-based MIP/IrO_2_/indium tin oxide (ITO) working electrode system. The incorporation of IrO_2_ was beneficial due to its high solubility and chemical stability, which improved the analyte reduction capabilities, while it produced an easily observable color change upon redox activity. As a note, the extraction of the template during sensor preparation was achieved rapidly, using 6 cycles of CV sweeping from −0.5 to 1.5 V in a methanol/acetic acid solution, followed by washes with distilled water. Therefore, the electrochromic nature of the sensor could be used for both quantitative and qualitative detection [16]. However, the use of heavy metals remains problematic because of their limited availability and potential toxicity [17,18]. To address these concerns, several strategies have been developed, particularly through the use of glassy carbon electrodes (GCEs). Although GCEs are chemically inert under diverse conditions and provide a wide electrochemical window, their relatively low electrical conductivity necessitates surface modification to achieve efficient sensing [15,19]. An earlier strategy for imidacloprid (IMD) detection in fruit samples using MIP-based neonicotinoid sensor was modifying the GCE with reduced graphene oxide (rGO) to enhance the sensor performance. Template molecules were removed using 0.5 M HCl and water washes over 60 min. The incorporation of a poly(o-phenylenediamine) (PoPD) MIP layer was shown to further improve the reproducibility of the measurements performed by linear sweep voltammetry (LSV) [15]. While DPV is widely employed due to its high sensitivity, LSV has also gained attention as a complementary technique, offering simpler instrumentation and reliable current–potential responses [14,20]. LSV operates by measuring the current at a working electrode while the potential is varied linearly with respect to a reference [20]. The presence of thiamethoxam (TMX) in grain was investigated by LSV, using a p-vinylbenzoic acid (p-VBA) derived MIP thin film coating on a graphene (GN)-modified GCE. The thin film architecture facilitated template removal and improved reusability while maintaining high sensitivity, selectivity, reproducibility, and accuracy [21]. An analogous sensing system was employed for the detection of IMD in rice. The MIP immobilization onto a GN nanosheet-modified GCE through π-π interactions improved the uniformity of the MIP layer formation [22]. Template extraction was achieved in 2 CV cycles, scanning from −0.2 to 1.4 V in a 0.1 M phosphate-buffered solution at pH 7.2. Both the linear working range and limit of detection (LOD) were comparable to those previously reported [21,22].

Other carbon-based working electrode modifications have been investigated with the aim of further improving sensitivity. Ghodsi and Rafati employed SWV to quantify IMD in water samples using a TiO_2_ nanoparticle-modified GCE. This modification improved the electrocatalytic reduction of IMD, increased stability over repeated measurements, and decreased the environmental impact and cost. The sensor also included the first application of a poly(levodopa) MIP for neonicotinoid detection, which contributed to a rapid response, a wide linear range of 2–400 µM, and good repeatability [23]. Li et al. introduced a more elaborate DPV-based strategy for IMD detection in vegetable samples, such as cabbage and chili [24]. They first modified the GCE working electrode surface with a Pt-In nanocomposite film, followed by electropolymerization of o-aminophenol doped with bromophenol blue, using a 4-tert-butylcalix[6]arene-IMD supramolecular inclusion complex as a template to assemble the MIP. While the presence of the 4-tert-butylcalix[6]arene binding sites enhanced porosity, leading to an increase in IMD binding sites for specific recognition, the novel strategy employing bromophenol blue and the Pt-In nanocomposite improved signal amplification and led to more efficient catalysis. A linear working range of 2.0 × 10^−10^–5.0 × 10^−8^ M and an LOD of 1.2 × 10^−11^ M were obtained [24].

**Figure 3:**
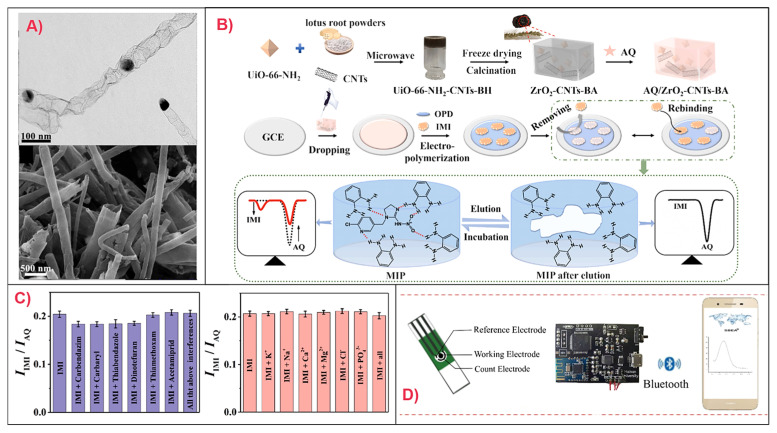
Development of selected MIP-based voltammetric sensors for neonicotinoid detection. (**A**) TEM (top) and SEM (bottom) images of Co/Mo_2_C/N-CNTs for the development of an PoPD MIP-based DPV sensor for IMD. Reproduced from Feng et al. (2025) [26] under the terms of the Creative Commons Attribution (CC BY) license. (**B**) Workflow for PoPD MIP/AQ/ZrO_2_-CNTs biomass-derived aerogel/GCE production and corresponding DPV measurements for IMD detection; (**C**) Effects of interferents on ratiometric current intensities at fixed IMD concentration for the sensor in (**B**). Reproduced with permission from Li et al. (2024) [29] © 2024 Elsevier B.V. (**D**) Design of a chitosan/GDA MIP/AuNPs/rGO/Screen-printed carbon electrode, paired with a miniaturized electrochemical workstation and smartphone platform connected via Bluetooth for a rapid, easily portable TMX sensor. Reproduced from Peng et al. (2021) [33], under the terms of the Creative Commons Attribution License.

Recently, a DPV-based sensor was developed for ACT detection in fruits such as apples and watermelons. In this sensor, a carbon paste electrode modified with multi-walled carbon nanotubes (MWCNTs), which enhanced conductivity, was used as the working electrode. Polymerization of 4-vinylpyridine with ethylene glycol dimethyl acrylate (EGDMA) as a cross-linker formed the MIP, producing a simple, accurate, and cost-effective sensor with high sensitivity and minimally affected signal in the presence of interferents [25]. Another DPV-based sensor was designed for IMD detection in tea using a GCE [26]. The GCE was modified with Mo_2_C nanodots anchored on Co-containing N-doped carbon nanotubes (N-CNTs), followed by modification with an MIP layer (Figure 3A). The template was removed using a methanol/acetic acid (9:1) solution over 16 min. This design resulted in highly exposed catalytic active sites and enhanced electrical conductivity while maintaining structural stability and strong binding affinity for the target analyte under electrochemical conditions. The sensor exhibited a linear range of (0.1–100) × 10^−6^ M and an LOD of 0.033 × 10^−6^ M. Although the sensitivity was relatively low compared to other designs, good selectivity in the presence of ionic and neonicotinoid interferents was achieved, and the high stability through repeated uses was advantageous for decreasing environmental impact and for on-site detection [26].

Although the use of MIPs has been shown to reduce signal interferences from other pesticides or common ions, using an internal reference to develop a ratiometric sensor can further improve the sensor performance. For instance, a ratiometric DPV-based sensor has been reported for detecting IMD in fruits. In this sensor, gold nanoparticles (AuNPs) were first electrodeposited onto a GCE surface, followed by self-assembly of the 6-(ferrocyanyl)hexanethiol (FcHT) internal reference and deposition of a PoPD MIP layer. The FcHT provided a stable signal in the presence of varied concentrations of IMD (signal on-off) in real samples and successfully minimized interference effects, and improved the sensitivity and accuracy of the sensor [27]. A similar ratiometric DPV strategy was developed for ACT detection in cowpeas. In this design, a magnetic GCE was used to improve the attachment of silver nanoparticle (AgNP)-modified Fe_3_O_4_ nanocomposites, which governed electrical conductivity, catalytic activity, and ratiometric detection. The system also employed a polydopamine (PDA)-based MIP layer, which enhanced the sensor sensitivity and rapid sensing due to high affinity and surface area for analyte binding [28].

Ratiometric MIP-based voltammetric sensors for neonicotinoid detection using sustainable carbon sources have also been developed. A recent study reported an IMD ratiometric DPV-based sensor for fruit and vegetable analysis, which utilized a UiO-NH_2_-CNTs (carbon nanotubes)-biomass hydrogel derived from lotus root powder. A working ZrO_2_-CNTs aerogel-modified GCE was developed and combined with a PoPD MIP. In this sensor, anthraquinone (AQ) served as the internal reference. The high surface area and low density of the aerogel provided a large number of analyte binding sites. While the ZrO_2_ acted as the catalyst, the CNTs provided structural support for the system and improved electrical conductivity. This design achieved an LOD of 0.014 µM with good reproducibility and selectivity in the presence of interferents (Figure 3B,C) [29]. Another ratiometric DPV-based sensor was developed for dinotefuran (DNF) detection in agricultural food products and insecticidal spray. A GCE working electrode was modified with a β-cyclodextrin/activated mung bean derived carbon (β-CD/AMBC-3) highly porous composite. Template removal was achieved using a methanol/acetic acid (8:2) bath for 10 min. The AMBC-3 improved electrode conductivity and β-CD immobilization, and consequently signal intensity. A catechol and poly(thionine) co-MIP film was then electrodeposited onto the electrode surface, with the poly(thionine) serving as an internal reference for ratiometric detection. The use of a copolymer-based MIP also improved binding site selectivity, yielding an LOD of 0.016 µM [30]. The durability of the sensors was high under careful storage conditions [29,30].

The simultaneous detection of multiple analytes is also advantageous for practical applications. For instance, a DPV-based MIP sensor for IMD and bensulfuron-methyl detection in water was developed, due to the non-overlapping redox peaks of the two analytes. A GCE was modified with MWCNTs to increase the binding surface area and conductivity. The presence of carboxyl groups allowed functionalization with a dual-template MIP (DMIP) involving PoPD and poly(thionine) for recognition of the non-electroactive bensulfuron-methyl. The method was found to have high sensitivity, good selectivity and stability, and a rapid response over a wide linear range [31].

A challenge for the development of rapid and user-friendly MIP-based electrochemical sensors for neonicotinoid detection is the extensive pre-treatment required for solid food samples [32,33]. To address this, research has focused on developing less destructive, portable detection systems. For example, DPV-based detection of TMX and IMD detection in mango, cowpea, and water was developed using a screen-printed carbon electrode modified with rGO and AuNPs, followed by modification by a chitosan/glutaraldehyde (GDA) cross-linked MIP. This strategy created affordable, rapid, and reproducible detection that was unaffected by a range of interferents. The use of chitosan promoted stronger analyte binding. Moreover, integration of a smartphone application connected via Bluetooth was advantageous for ease of operation and data collection. The instrument was designed to be lighter, smaller, and thus more portable compared to conventional electrochemical workstations (Figure 3D) [32,33].

Cyclic voltammetry (CV), which can monitor both oxidation and reduction processes, can also be used to develop voltammetry-based sensors [13]. An IMD microneedle-based sensor was applied for honey analysis using CV and DPV. Notably, comparable LODs and linear working ranges were obtained by both techniques. The method did not require extensive sample preparation. The microneedle was composed of a CNT/cellulose nanocrystal (CNC) composite, and polyaniline (PANI) was used to achieve the MIP biomimetic receptor film. While the CNTs allowed for a high electrocatalytic surface area, PANI promoted conductivity and was favorable due to its simple preparation, environmental stability, and adjustable properties. Additionally, the microneedle showed excellent reusability, portability, accuracy, and cost-effectiveness [34].

### 2.2. Potentiometric Sensors

Compared to voltammetry-based sensors, potentiometry-based MIP sensors have been less employed for neonicotinoid detection. Nonetheless, potentiometry can be used to develop sensors with high sensitivity, selectivity, accuracy, and low cost [35]. Unlike voltammetry, which measures current responses to applied potentials, potentiometry measures changes in the potential difference between a working and reference electrode under zero-current conditions, providing a simple and robust readout of analyte concentration [13,36]. Several potentiometric DNF sensors have been developed in one study for cucumber and soil analysis. They used MIP-functionalized PVC membranes to clip onto the end of a glass electrode. It was found that washed acrylamide (AM) MIPs with EGDMA cross-linkers had the highest binding affinity and sensitivity compared to unwashed, methacrylic acid (MAA)-based MIP or carboxylated PVC variants. However, all the sensors had straightforward fabrication and showed good precision, rapid response, and minimal sample pre-treatment [35]. Similarly, Kamel and Abd-Rabboh detected IMD in pesticide samples using a rGO-modified GCE coated with MAA/EGDMA MIP beads and spread across a PVC ion-selective electrode (ISE) membrane [37]. The sensor showed a linear range of 0.5 µM–1.0 mM and an LOD of 0.2 µM using small sample volumes and in complex matrices. The sensor was also portable, with high stability and durability [37]. Thus, while potentiometric MIP sensors are less common than their voltammetric counterparts, they can provide a reusable, cost-effective platform well-suited for portable, field-based, and real-time monitoring applications.

### 2.3. Photoelectrochemical Sensors

MIP-based photoelectrochemical (PEC) sensors for neonicotinoid detection have attracted attention due to their extremely low LODs, making them useful for trace analysis [38]. PEC sensors utilize photoactive materials to convert light into an electric current, which can be affected by the presence of an analyte [39]. Research for this method has focused on electrode heterojunction modification to enhance photoelectric responses by accelerating charge separation and electron transfer. For example, a clothianidin (CLT) PEC sensor was developed for water analysis. A fluorine-doped tin oxide (FTO) working electrode was modified with a WO_3_/CdS heterojunction and an electropolymerized PoPD MIP, resulting in a highly sensitive, selective, and cost-effective sensor with an LOD of 0.4 nM and a wide linear working range of 1.0 nM–5.0 µM [40]. Conversely, TMX concentrations were determined in environmental water samples using a PEC sensor using a ZnO/Bi_2_O_3_/Bi_2_S_3_ heterojunction on an FTO working electrode. An MIP prepared with chitosan and a GDA cross-linker resulted in high sensitivity, selectivity and accuracy, in addition to minimal interference effects. The use of ZnO improved light-to-current conversion efficiencies and separation of the redox active centers within the semiconductor, resulting in an LOD of 3.32 × 10^−13^ M and a linear working range of 7.0 × 10^−13^–7.0 × 10^−10^ M. However, the sensor fabrication was complex, and it had low stability in acidic environments [41]. Therefore, further advances in electrode architecture and material robustness are needed to reach the full potential of PEC-MIP sensors. Overall, PEC sensors offer a powerful complement to voltammetric and potentiometric methods, particularly for environmental monitoring applications where ultra-trace detection and high sensitivity are critical.

### 2.4. Electrochemical Impedance-Based Sensors

MIP-based electrochemical impedance sensors have also been developed for neonicotinoid detection. Capacitive sensors detect analyte presence due to changes in capacitance within a dielectric material [42]. In one example, an automated flow injection system was successfully integrated for capacitance-based detection of IMD in water samples, using a MAA/EGDMA MIP and electropolymerized tyramine on a gold working electrode. The sensor demonstrated high accuracy, precision, sensitivity, and reproducibility. This reusable sensor required only small sample volumes [43]. Finally, electrochemical impedance spectroscopy (EIS) is a powerful technique that monitors changes in the interfacial properties of an electrode by applying an AC voltage and measuring the resulting impedance across a range of frequencies [44]. Unlike voltammetric methods, which monitor redox processes, or potentiometric sensors, which rely on equilibrium potential shifts, impedance-based detection is highly sensitive to surface conductivity properties [13,36,44]. An EIS-based sensor was applied to the determination of IMD in water samples using an electropolymerized PoPD MIP on a WO_3_/MoS_2_ nanosheet-modified FTO working electrode. In this design, MoS_2_ enhanced conductivity, while WO_3_ contributed to high electron mobility and had the advantages of abundance, low cost, high stability, and an easily modifiable structure. The sensor showed high sensitivity and selectivity in the presence of interferents [45]. Overall, impedance-based MIP sensors provide attractive features such as reusability, integration with flow systems, and efficient detection in small sample volumes. However, their detection limits and dynamic ranges remain less competitive than those of PEC or voltammetric sensors. With further refinement in electrode design and signal amplification strategies, impedance-based platforms could emerge as a valuable complementary tool for real-time, reusable detection of neonicotinoids.

### 2.5. Electrochemical Sensor Limitations and Future Research

Table 1 summarizes the various MIP-based electrochemical sensors for neonicotinoid detection. Among the different electrochemical sensors, PEC sensors generally achieved the lowest LODs, while voltammetric sensors provided the greatest balance between low LODs and broad linear ranges of detection, particularly when employing DPV.

Table 2 presents representative examples of electrochemical sensors for neonicotinoid detection, highlighting their response times, selectivity, stability, and applications in measuring real samples. Generally, good recovery was found in real samples across different detection methods, and the sensors had excellent stability for at least 2 weeks or sensor reuse. The sensors provided rapid response times following sample incubation and showed generally high selectivity towards the target analytes. Voltammetric MIP sensors also have the potential for miniaturization and integration with portable, low-cost readouts, such as microneedles and smartphone-based detection systems. Moreover, significant work has been performed to develop modified electrodes for voltammetry-based sensors from sustainable materials and for ratiometric detection strategies, reducing environmental impact and improving sensor reliability, respectively. Although potentiometric, PEC or impedance-based MIP sensors have yet to be as widely developed for these goals, these methods show significant promise in terms of sensitivity, selectivity, efficiency and reusability. Future progress in surface engineering and device integration may therefore expand their applications for practical neonicotinoid monitoring.

## 3. Optical Sensors

Optical sensing methods employing MIPs for detecting neonicotinoids have gained significant attention due to their inherent advantages of high sensitivity, rapid response, minimal sample preparation, and potential for portable and cost-effective instrumentation. These methods typically monitor changes in optical properties, such as absorbance or color changes, luminescence intensity, wavelength or lifetime shifts, refractive index variations, scattering and diffraction phenomena upon analyte interaction [40,46,47]. Using MIPs further enhances sensor selectivity and specificity, ensuring robust and accurate measurements even in complex sample matrices [48,49]. Among the most widely studied optical detection modalities are electrochemiluminescence (ECL), fluorescence, and surface-enhanced Raman scattering (SERS). Other approaches, including colorimetry/UV-Vis absorbance, surface plasmon resonance (SPR/LSPR), chemiluminescence, photonic crystal sensors, and fiber-optic waveguide platforms, have also been reported in MIP-based sensing [47,49,50,51,52]. Each of these methods offers unique benefits in terms of sensitivity, selectivity, and adaptability to real-world applications. Recent innovations have focused on combining MIPs with advanced novel nanomaterials and nanostructures, which offer larger surface areas, improved analyte binding, and amplified optical signals [50,53]. This section will examine recent advancements in optical MIP-based sensors, highlighting their mechanisms, performance characteristics, and practical applicability for detecting neonicotinoid residues in environmental and food samples.

### 3.1. Electrochemiluminescence (ECL) Sensors

ECL sensors have emerged as a powerful analytical technique for detecting neonicotinoids, combining the advantages of electrochemical and optical methods. ECL sensors operate by measuring photons emitted from electrochemically generated excited states of luminescent materials, eliminating the need for an external light source [54]. This unique mechanism results in low background noise, high sensitivity, and excellent selectivity [54,55]. The integration of MIPs with ECL sensors further improves their performance in neonicotinoid detection. Recent advancements in ECL-MIP sensors for neonicotinoids have focused on designing novel luminophores, developing signal amplification strategies, and incorporating nanomaterials to enhance sensor performance [51,54,55].

Ma et al. reported a highly sensitive and stable ECL sensor for detecting trace amounts of IMD in plant-derived foods, using ultrafine mixed-valence cerium-metal–organic framework (Ce-MOF) nanowires [55]. These Ce-MOF nanowires, synthesized via a micelle-assisted biomimetic route, exhibited significantly enhanced conductivity and water stability compared to conventional MOFs. Integration of these nanowires into the luminol-hydrogen peroxide ECL system facilitated a dual-route self-circulating catalytic amplification mechanism, considerably increasing the ECL intensity. Molecular imprinting with o-phenylenediamine (o-PD) as the functional monomer created selective recognition sites IMD, resulting in a linear detection range of 2–120 nM and a low LOD of 0.34 nM. This strategy not only improved sensor stability but also simplified electrode modification procedures due to facile nanowire immobilization. Despite these advancements, achieving broader detection ranges and improved selectivity in complex environmental and food matrices still posed challenges. To address these limitations, Tang et al. proposed an ECL sensor combining lanthanide-doped upconversion nanoparticles (UCNPs) functionalized onto zeolite imidazolate framework-8 (UCNPs@ZIF-8), coupled with molecular imprinting for the selective detection of IMD [51]. The sensor was fabricated by first immobilizing UCNPs onto ZIF-8 particles to form UCNPs@ZIF-8. The resulting composite was then used to electropolymerize the imprinted polymer layer on a glassy carbon electrode in the presence of IMD as the template molecule. After polymerization, the electrode was rinsed with ultrapure water and immersed in methanol/water (1:1 *v*/*v*) for 10 min to elute the template molecules, exposing the IMD-specific recognition sites. The UCNPs significantly enhanced luminescent properties, while ZIF-8 provided structural stability and a high surface area for effective immobilization of UCNPs. This design markedly improved sensor selectivity and sensitivity, achieving an exceptionally wide linear range (3.9 × 10^−4^–3.9 × 10^3^ nM) and an ultra-low LOD (3.9 × 10^−5^ nM). The sensor delivered accurate and sensitive results for IMD in real food samples, highlighting its potential for field applications.

Further progress in improving sensitivity and selectivity was demonstrated by Cai et al., who developed an advanced dual-source amplified ECL sensor employing a multifunctional composite consisting of lanthanide-based metal–organic gels (Tb-Ru-MOG), cerium oxide nanoparticles (CeO_2_), and nitrogen-doped graphdiyne (N-GDY) [54]. The dual-source amplification originated from the intrinsic catalytic effect of CeO_2_ nanoparticles, which efficiently generate sulfate radical, and from the superior electron transfer capability of N-GDY. Molecular imprinting with bifunctional monomers further endowed the sensor with selective binding sites tailored specifically for IMD, facilitating precise analyte recognition even in the presence of structurally similar compounds. This design resulted in enhanced selectivity and robustness, allowing the sensor to achieve an impressive linear detection range (10–10,000 nM) and a competitive LOD of 1.37 nM. Additionally, the incorporation of graphdiyne significantly improved the conductivity and stability of the sensor, supporting potential field applications. Despite these advances, several challenges remain; similar to electrochemical sensors, ECL-based platforms can be susceptible to interference from complex matrices, requiring careful optimization of electrode surfaces and MIP formulations [54,55].

### 3.2. Fluorescence Sensors

Fluorescence-based sensors are highly valued for neonicotinoid detection, as they offer rapid response, high sensitivity, low detection limits, and easy integration into portable devices [47,48,56]. These sensors operate by monitoring changes in emission intensity, wavelength shifts, or fluorescence lifetime induced by analyte interactions, which results in either fluorescence quenching, enhancement, or spectral shifts [47,56,57,58]. Although traditional fluorescence sensors provide simple and fast analysis, their selectivity can be limited, especially in complex matrices, which are prone to interference from structurally similar compounds [48,56]. To address this, MIP-based fluorescence sensors have become increasingly promising. By merging the sensitivity of fluorescence detection with the selectivity of molecular imprinting, fluorescent MIP-based sensors achieve precise detection of low concentrations of target analytes in environmental samples [47,48,56]. Recent advancements have leveraged innovative fluorophores, including quantum dots (QDs), graphene quantum dots (GQDs), carbon dots (CDs), polymer dots (PDs), organic dyes, and metal–organic frameworks (MOFs), to mitigate common limitations such as photobleaching, low target selectivity, and poor reproducibility [47,48,49,57,58,59,60].

Among the promising fluorophores explored, carbon-based materials have received considerable attention due to their high quantum yields, good biocompatibility, and low toxicity [47]. Shirani et al. developed a MIP-based fluorescence sensor embedding silane-doped carbon dots (Si-CDs) for the selective detection of ACT [49]. This sensor utilized polymerization to form a MIP-coated Si-CD composite, exhibiting a selective fluorescence quenching response towards ACT. Under optimized conditions, the sensor demonstrated excellent sensitivity, achieving a detection limit as low as 2 nM, with a linear detection range between 7 and 107 nM. The integration of Si-CDs within a silica matrix enhanced the sensor’s stability and selectivity, rendering it suitable for practical analysis of ACT in environmental samples.

In a related study, Ghani et al. evaluated PDs and carbon quantum dots (CQDs) for MIP-based fluorescence sensing of ACT [60]. The PD-based sensors showed superior analytical performance compared to CQD-based systems, exhibiting an ultra-low detection limit and a wide dynamic detection range [60]. These polymeric dots demonstrated higher quantum yields and lower aggregation-induced fluorescence quenching, indicating significant potential for highly sensitive pesticide detection in complex matrices such as water and agricultural products. To confirm its applicability, the study validated the developed sensor using five real matrices: sea, well, river, and tap water samples, as well as apples. The sensor demonstrated excellent precision and recovery, consistent with ion mobility spectroscopy results. These MIP-functionalized nanoparticles showed strong and selective responses to ACT over structurally similar analogs, unlike non-imprinted counterparts (NIPs). Further advancement was presented by Pan et al., who developed nitrogen-doped CDs integrated into inverse opal photonic crystal (IOPC) hydrogel structures, resulting in improved analyte accessibility and enhanced fluorescence response [47]. The hydrogel was produced via sol–gel polymerization, after which the imprinted matrix was immersed in methanol/acetic acid (9:1 *v*/*v*) for 10 min to extract the template molecules, forming well-defined IMD cavities. This architecture provided efficient mass transfer channels and rapid response times (equilibrium within 20 min), alongside excellent stability, selectivity, and reusability. The resultant sensing strips offered reliable detection of IMD in food matrices with a detection limit of 0.25 nM and a linear response ranging from 0.39 to 196 nM. This sensor demonstrated practical applicability, validated through comparison with high-performance liquid chromatography (HPLC) results.

**Figure 4:**
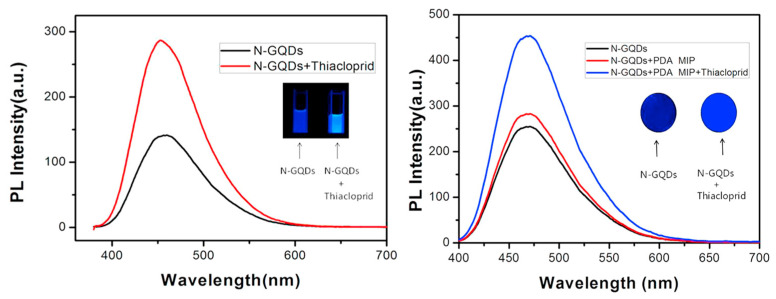
Representative MIP-based fluorescence sensors for selective neonicotinoid detection. PL emission spectra of N-GQDs before and after addition of THC (**left**), and corresponding response on MIP-coated test strips (**right**). Notable PL enhancement and visually distinguishable blue fluorescence under 365 nm UV confirm high specificity and usability of the sensor platform. Reproduced with permission from Liu et al. (2018) [57] © 2018 Elsevier B.V.

In parallel, GQDs and up conversion nanoparticles (UCNPs) have gained traction due to their exceptional photostability and low photobleaching [59]. Liu et al. designed a portable, test-strip-based fluorescence sensor employing nitrogen-doped graphene quantum dots (N-GQDs) and PDA-based molecular imprinting for selective thiacloprid (THC) detection [57]. GQDs represent a novel class of fluorescent nanomaterials valued for their chemical stability, facile synthesis, low cytotoxicity, and excellent biocompatibility. However, GQD surfaces often allow limited modification and exhibit low quantum yield, which limits their broader utility. To overcome this, Liu et al. have employed nitrogen doping to enhance photoluminescence (PL) efficiency and stability [57]. The sensor was prepared by immersing filter paper in a diluted N-GQD solution (0.1 g L^−1^) to provide a fluorescent layer. After drying, the filter paper was immersed in a solution containing dopamine (DA) and THC in Tris buffer (10 mM, pH 8.5). In this solution, DA underwent self-polymerization to form PDA-MIP coating layer. DA, a neurotransmitter capable of oxidative self-polymerization under mild conditions served as both the functional monomer and crosslinker to form a MIP film on the strip surface. After polymerization, unreacted DA was removed, and the modified PDA-MIP strip was thoroughly rinsed with ultrapure water and subsequently immersed in 5 wt% acetic acid/SDS solution for one hour to extract the THC template molecules. This process produced well-defined recognition cavities that are complementary to the target molecule. This straightforward, paper-based design represented a novel approach to neonicotinoid detection, providing a low-cost, portable, and easily visualized sensing platform. The resulting test strips offered a linear detection range of 396–39,600 nM with a detection limit of 119 nM. As shown in Figure 4, the PL of the MIP sensor strip clearly increased in the presence of THC under UV irradiation, confirming its visual selectivity and ease of practical use [57]. The integration of nitrogen doping with PDA imprinting enhanced fluorescence efficiency and molecular recognition, producing well-defined binding sites that substantially improved the sensor’s reproducibility and selectivity compared to conventional QD-based systems. Yu et al. further improved fluorescence detection sensitivity by employing molecularly imprinted UCNPs encapsulated within a silica shell for ACT detection [59]. The sensor exploited a fluorescence-quenching mechanism based on photo-induced electron transfer, demonstrating high specificity with an imprinting factor of 7.84. This MIP-coated UCNP platform provided an excellent linear relationship within the concentration range of 89.8–3592 nM, achieving a low detection limit of 37.3 nM. Its robustness and reproducibility were validated by successful applications to real food matrices, emphasizing the practicality of UCNP-based sensing systems for food safety monitoring.

MOFs have also recently been integrated with MIPs due to their high quantum yield and narrow emission band. Cao et al. developed a smartphone-enabled fluorescence sensing platform using europium-based MOFs (Eu (BTC)-MPS) functionalized with MIP layers for CLT detection in vegetables [58]. The sensor exhibited high selectivity attributed to effective molecular imprinting on MOF surfaces, achieving a wide linear response (40–40,048 nM) with a LOD of 16 nM, suitable for smartphone-based analysis. The sensing mechanism, based on fluorescence quenching through the inner filter effect (IFE), allowed for rapid on-site detection and straightforward integration with smartphone-based imaging technology, significantly enhancing accessibility and practical applicability for consumer-level analysis.

Complementing this, Xie et al. introduced magnetic core–shell fluorescence sensors (Fe_3_O_4_@SiO_2_@MIPIL) combining molecular imprinting, fluorescence analysis, and ionic liquid functional monomers for IMD determination [48]. The Fe_3_O_4_@SiO_2_@MIPIL sensor was fabricated by a stepwise procedure involving silica coating, 3-(Trimethoxysilyl)propyl methacrylate (TMOPMAS) surface functionalization, and ionic liquid-based molecular imprinting to form a well-defined magnetic core–shell architecture tailored for selective IMD recognition. This multifunctional composite allowed for rapid analyte separation and analysis within one minute, showcasing a linear detection range (1–1000 nM) and a low detection limit (0.3 nM). The magnetic separation capability simplified the sample preparation and extraction process, substantially reducing analytical complexity and enhancing the sensor’s practical utility in environmental and food safety applications.

Despite this substantial progress, fluorescence-based MIP sensors still encounter several challenges. Issues such as sensor photostability, environmental adaptability, fluorescence quenching interferences from complex matrices, and limitations in large-scale sensor fabrication remain critical [47,56,58]. Future research directions could involve exploring hybrid or dual-mode fluorescence systems, incorporating ratiometric detection mechanisms for enhanced accuracy, and expanding sensor integration into portable and wearable devices [46,47,57,58,59].

### 3.3. Ratiometric Fluorescence Sensors

Ratiometric fluorescence sensors provide a significant advantage over conventional single-emission fluorescence techniques by employing dual-emission systems: typically, one responsive to analyte concentration and another as an internal reference [61]. This strategy provides built-in calibration, enhances detection precision, and minimizes signal fluctuations due to environmental interferences or operator error, making it especially suitable for practical, on-site neonicotinoid detection in complex matrices [46,61].

**Figure 5:**
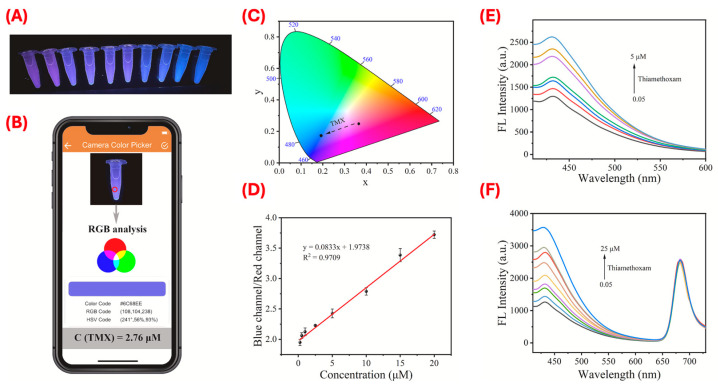
Ratiometric MIP-based fluorescence sensing for TMX detection and smartphone-enabled quantification. (**A**–**D**) Visual and analytical outputs of the smartphone-assisted dual-emission sensing system. (**A**) Fluorescence color change under 365 nm UV light with increasing TMX concentrations. (**B**) Real-time RGB analysis using a smartphone app. (**C**) CIE chromaticity coordinate corresponding to the fluorescence shifts. (**D**) Linear correlation between blue/red (B/R) fluorescence channel ratio and TMX concentration (0.125–20 µM). (**E**,**F**) Fluorescence responses of single-emission (**E**) and ratiometric dual-emission (**F**) B-CDs@MIPs/R-CDs sensors. The ratiometric sensor demonstrated enhanced sensitivity and a wider linear range. Reproduced with permission from Dai et al. (2023) [61] © 2023 Elsevier B.V.

Dai et al. developed a smartphone-enabled ratiometric fluorescence sensor specifically for visual detection of TMX [61]. This sensor comprised dual-color emission CDs: blue-emission CDs (B-CDs) and red-emission CDs (R-CDs) [61]. The B-CDs were coated with MIPs using a sol–gel method to form B-CDs@MIPs, which served as the analyte-responsive signal, while R-CDs functioned as the internal reference. The presence of TMX induced a visible shift in fluorescence color from red to blue, reflected by an increase in the fluorescence intensity ratio between blue and red emissions. This dual-emission approach greatly reduced signal fluctuations caused by environmental factors and enhanced the sensor’s reliability. The sensor demonstrated a detection limit of 13.5 nM by fluorescence spectroscopy and 70.1 nM visually via smartphone imaging. It also exhibited strong selectivity, effectively distinguishing TMX from other structurally similar pesticides, ions, and other environmental interferents. These attributes make this sensor highly suitable for accurate and reliable field detection of TMX residues in agricultural and environmental samples. The performance and visual output of this sensing platform are illustrated in Figure 5, which showcases both the smartphone-enabled RGB analysis and the dual-emission fluorescence response. Wei et al. introduced a novel ratiometric fluorescence sensor based on molecularly imprinted multilevel mesoporous silica (MIFP-SiCQDs@CdTe QDs) for highly sensitive detection of IMD [46]. This design featured dual-emission quantum dots: silicon-carbon quantum dots (SiCQDs), which emit blue fluorescence, acted as a stable internal reference, while CdTe QDs emitting red fluorescence served as the analyte-responsive signal.

Crucially, these QDs were strategically anchored into multilevel mesoporous silica through a post-imprinting modification method, enhancing sensor stability, improving analyte mass transfer, and preventing quantum dot leakage during template removal. The developed sensor displayed an outstanding linear detection range of 19.6 to 1956 nM with a low detection limit of 13.9 nM. Furthermore, its excellent selectivity against structurally similar pesticides and common environmental interferents, along with demonstrated successful application to real food and environmental samples, underscored its practical potential.

### 3.4. Surface-Enhanced Raman Scattering (SERS) Sensors

Surface-enhanced Raman scattering (SERS) sensors present a highly effective analytical method for detecting neonicotinoid insecticides due to their exceptional sensitivity, molecular specificity, and ability to provide detailed structural fingerprint information [50,62]. SERS techniques detect unique vibrational signatures of analytes that are localized within electromagnetic hotspots on plasmonic metal surfaces, typically gold (Au), silver (Ag), or copper (Cu) [63,64]. This localization significantly amplifies Raman scattering signals, facilitating trace-level detection [63]. Despite their remarkable advantages, SERS sensors face challenges, such as matrix interference, where signals from interfering substances in complex matrices can obscure the analyte’s specific signal [64]. To overcome this limitation, research has focused on modifying SERS substrates by immobilizing MIPs to enhance selectivity and reproducibility. This allows the sensors to selectively trap the target analytes near the electromagnetic hotspots, amplifying the SERS signal [64]. Consequently, MIP-based SERS sensors demonstrate considerable potential for robust, specific, and sensitive neonicotinoid detection in environmental and food samples [50,62].

Tang et al. advanced this concept by developing a three-in-one MIP-based electrochemical SERS (MIP-EC-SERS) sensor specifically tailored for detecting ACT residues in vegetables [64]. This sensor employed an electropolymerized PDA layer with molecularly imprinted cavities on AuNPs/ITO electrodes, which facilitated selective capture of ACT molecules. By applying a controlled electrochemical potential (+0.3 V), the researchers significantly enhanced interaction between ACT and the SERS-active surface, resulting in substantial signal amplification. The sensor demonstrated an excellent linear detection range from 10 nM to 20,000 nM, with an impressively low detection limit of 3.2 nM, which is 13.6 times lower than LOD of MIP-SERS (43.5 nM). Moreover, the practical applicability of this sensor was validated through accurate detection in vegetable samples, highlighting its robust potential for reliable and cost-effective food safety monitoring.

**Figure 6:**
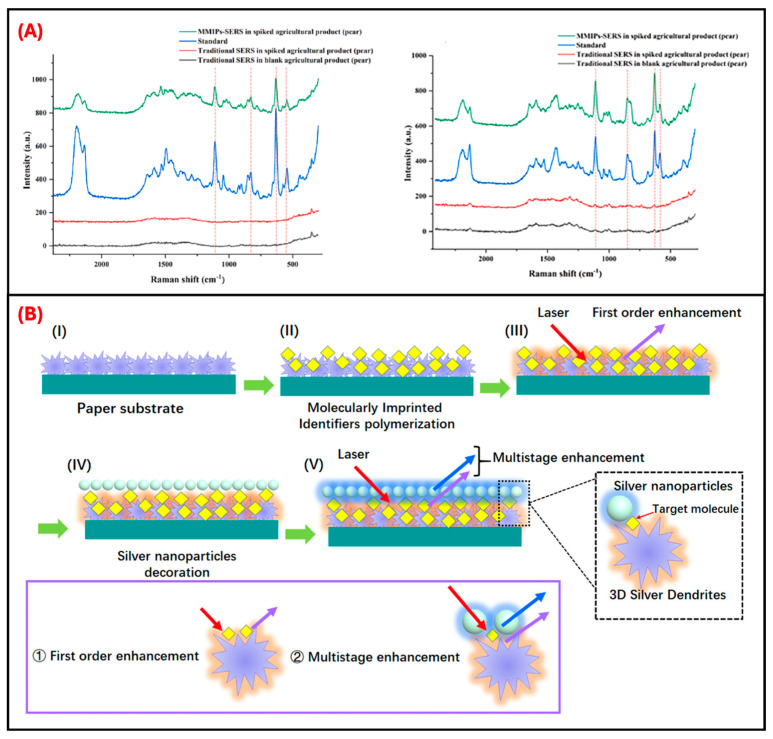
MMIP-SERS detection of neonicotinoids in agricultural samples and enhancement mechanism via 3D silver dendrites. (**A**) Representative Raman spectra for the detection of ACT (left) and THC (right) in spiked pear samples. The MMIP-SERS sensor reveals clear, target-specific peaks in real matrices, unlike traditional SERS methods. Reproduced with permission from Cao et al. (2024) [65] © 2023 Elsevier B.V. (**B**) Schematic illustration of multistage electromagnetic hotspot generation through 3D silver dendrite growth, molecular imprinting, and AgNP decoration. Enhanced field overlap and hotspot density support significant SERS signal amplification. Reproduced with permission from Zhao et al. (2020) [50] Copyright © 2020 American Chemical Society.

One innovative strategy involved coupling magnetic molecularly imprinted polymers (MMIPs) with SERS, leveraging both high selectivity and easy sample handling. Cao et al. developed an MMIP-SERS sensor for the rapid analysis of ACT and THC in agricultural products. Magnetic nanoparticles served as supports for imprinting ACT-specific MIP layers, enhancing rapid adsorption (saturation in only 1 min), and allowing for simple magnetic separation of analytes from complex matrices [65]. Employing AuNPs as the SERS substrate, the system achieved LODs of 309 and 151 nM for ACT, and 144 and 94 nM for THC, in pear and peach samples, respectively, in peach and pear samples. The MMIP-SERS sensor demonstrated significantly enhanced Raman signal intensity and minimized background interference in spiked fruit samples compared to conventional SERS, underscoring the platform’s superior specificity and practical applicability. As illustrated in Figure 6A, the MMIP-SERS spectra clearly reveal characteristic peaks for ACT and THC in real agricultural matrices, highlighting the system’s interference resistance and field-deployable utility. Further extending the utility of magnetic MIP-SERS sensors, Bai et al. introduced a trifunctional Fe_3_O_4_@MIP nanocatalytic probe capable of specific recognition, catalytic generation of AuNPs, and magnetic enrichment for ultratrace DNF detection [66]. This nanoprobe enabled catalytic generation of AuNPs with strong SERS, resonant Rayleigh scattering (RRS), and surface plasmon resonance (SPR) signals. Binding of DNF significantly reduced catalytic activity, decreasing SERS signals linearly and allowing sensitive detection with an extremely low LOD of 0.009 nM. The sensor exhibited excellent enrichment (100-fold), selectivity, and rapid analyte separation through magnetic enrichment, greatly simplifying sample handling and detection workflows for practical applications.

Advanced plasmonic structures have also been explored for enhancing SERS sensor performance. Zhao et al. introduced a paper-based multilayer plasmonic SERS platform for highly sensitive neonicotinoid detection [50]. The substrate comprised three-dimensional silver dendrites (SDs), electropolymerized molecular identifiers (EMIs), and AgNPs in a unique sandwich hybrid architecture, effectively creating multiple hotspots and enhancing SERS sensitivity through electromagnetic coupling amplification. EMI films were prepared by electropolymerizing pyrrole in the presence of IMD as the template. The reaction was carried out in a deoxygenated electrolyte (purged with nitrogen gas) containing pyrrole (0.5 mM,) KCl (0.1 M), PBS (pH 6.86), and IMD (10 mM). Electropolymerization was then performed using cyclic voltammetry from −1.3 to +1.0 V at 50 mV s^−1^ for 15 cycles. Following polymerization, template molecules were extracted by applying 1.3 V in 0.2 M K_2_HPO_4_ for 20 min to yield clean recognition sites on the conductive paper. This multilayer plasmonic strategy creates multistage hotspot enhancement near the specific recognition site via synergistic electromagnetic field overlap (Figure 6B). This approach enabled achieving outstanding sensitivity for IMD, reaching an ultralow detection limit of 0.110 nM, significantly surpassing conventional single-layer SERS substrates. The use of EMI layers specifically facilitated selective target molecule capture and enrichment, demonstrating significant advantages for real-time, rapid neonicotinoid monitoring in complex matrices.

Chen et al. reported a novel dual-mode catalytic SERS/RRS sensor using nanopalladium (Pd) MIPs for highly sensitive and selective detection of DNF [63]. The Pd@MIP nanoprobe exhibited dual functionalities, including highly specific molecular recognition and catalytic activity toward AuNP formation, where the interaction with DNF improved the catalytic effect by coupling electrons on the nanosurface with π-electrons, resulting in strong and distinct Raman and Rayleigh scattering signals. The integration of Pd nanoparticles significantly amplified the electromagnetic field effects, producing sensitive detection limits (0.03 nM for SERS and 0.06 nM for RRS) and robust analytical reproducibility. This study effectively demonstrated the potential for dual-mode MIP-SERS platforms to achieve reliable and sensitive trace analysis, suitable for environmental monitoring and food safety applications. Shi et al. developed a bifunctional MIP nanosensor capable of catalytically amplifying SERS signals via gold nanoparticle generation, utilizing α-methacrylic acid-based MIPs specifically designed for ACT [62]. This strategy integrated both RRS and SERS modalities, resulting in an exceptionally low detection limit of 0.0001 nM for SERS. The nanocatalytic amplification approach significantly boosted sensitivity, allowing for ultrasensitive quantitative detection of ACT in complex environmental samples. This dual-mode sensor effectively leveraged molecular imprinting specificity combined with the signal enhancement provided by nanocatalytic generation of AuNPs, illustrating substantial improvements in sensitivity and practicality.

Ju et al. addressed challenges associated with matrix interference by combining molecularly imprinted solid-phase extraction (MISPE) with SERS technology for nitenpyram (NIT) detection in fruits [67]. This system employed precipitation-polymerized MIPs as selective adsorbents, achieving rapid analyte adsorption (within 10 min) and large adsorption capacities. Under optimized conditions, the MISPE-SERS method achieved LODs of 1400 nM in pear, 2730 nM in peach, 517 nM in apple, and 1400 nM in tomato, demonstrating high sensitivity in complex food matrices. The incorporation of selective MISPE pretreatment substantially reduced matrix interference, thus improving the analytical accuracy and applicability of the SERS-based detection strategy in practical scenarios.

### 3.5. Optical Sensor Limitations and Future Research

The key MIP-based optical sensors for neonicotinoid detection discussed in this review are summarized in Table 3. SERS sensors typically achieve the lowest LODs due to intense electromagnetic enhancement effects that dramatically increase sensitivity. Fluorescence sensors, especially those employing ratiometric techniques, provided an excellent balance between sensitivity, selectivity, and operational simplicity, making them ideal for practical on-site applications such as smartphone-based detection platforms. ECL sensors uniquely combined the merits of optical and electrochemical detection, demonstrating strong analytical performance with broader linear ranges and low LODs due to dual-source signal amplification strategies and advanced nanocomposite materials. Additionally, significant research efforts have focused on sensor portability and user-friendliness by integrating with accessible technologies, such as paper-based substrates and magnetic extraction platforms [50,65]. These approaches substantially reduce the complexity of sample preparation and improve real-world applications [50,65]. In parallel, the growing use of sustainable and cost-effective materials like CDs and MOFs reflects the current trend towards environmentally friendly and economically viable sensor development [57,58]. Despite these advances, several intrinsic challenges remain that limit sensor reproducibility, long-term stability, and standardization across studies.

Beyond the platform-related issues, MIPs themselves present inherent limitations that influence optical sensor reliability and analytical accuracy. One major concern is template leaching and incomplete removal, which can introduce false-positive responses, elevated baselines, or reduced reproducibility, particularly at trace-level analyte concentrations. While several optical MIP studies have confirmed template removal via solvent extraction or electrochemical washing [50,58], complete elimination is not always achieved. For instance, the Eu (BTC)-MPS@MIP fluorescence sensor maintained about 85% of its initial signal after four reuse cycles, suggesting gradual site degradation or residual template entrapment [58]. Similarly, certain ECL-based systems exhibited partial signal loss after repeated binding-elution operations [51,54], emphasizing the need for sturdier polymer architectures and optimized regeneration conditions. Another persistent issue is binding-site heterogeneity, arising from variations in monomer-template interactions and polymerization kinetics. Although most optical MIP sensors showed excellent selectivity in controlled conditions, validation in real matrices typically involved samples spiked only with the target analyte. In actual agricultural and environmental samples, however, multiple neonicotinoids or structurally related compounds may coexist and compete for binding, potentially causing recovery bias or signal suppression. Since such co-spiking or multi-analyte competition tests were rarely performed, the real-world impact of site heterogeneity remains poorly defined. Future studies should therefore include matrix-matched co-spike experiments to evaluate the selectivity and quantification accuracy of optical MIP sensors under realistic conditions. Batch-to-batch reproducibility also continues to pose practical challenges. Even when multiple cycles of synthesis produce consistent analytical responses [47,49,50,51,54,55,58,61,64], small differences in polymerization time, crosslinking ratios, or nanoparticle distribution can alter recognition-site density and sensitivity [47,49,65]. Establishing standardized imprinting protocols, together with advanced polymerization strategies such as surface imprinting or controlled polymerization, could improve structural homogeneity and ensure reproducible performance across production batches. Finally, consistent reporting of performance indicators remains uneven across studies. As shown in Table 3, not all reports provide full data on reproducibility, stability, reusability, or the imprinting factor (IF), which is a quantitative ratio that compares analyte binding between MIPs and their NIP counterparts. Adopting a unified set of performance descriptors, such as LOD, linear range, response time, selectivity, reproducibility, reusability, stability, and IF, would facilitate meaningful comparison and benchmarking among different optical MIP platforms.

Looking forward, future research should focus on three main directions. First, the standardization of evaluation metrics and synthesis protocols is essential to accurately compare MIP-based sensor performance across laboratories. Second, integrating optical MIPs with accessible readout systems such as smartphone imaging, paper-microfluidic formats, and magnetic SERS substrates will accelerate the translation of these sensors into field-deployable tools. Lastly, future research should continue exploring multimodal and integrated sensing systems that combine the high specificity of MIPs with portable detection methods like smartphone-based imaging, paper-based assays, or microfluidic devices [50,57,58,61,62,63]. Further improvements in sensor reproducibility, stability under field conditions, and the expansion of sensor capabilities to simultaneously detect multiple analytes are promising avenues that will lead to more profound investigation [48,50,57].

## 4. Miscellaneous Sensing Methods

Some researchers have developed MIP-based neonicotinoid sensors using analytical techniques beyond electrochemical or optical methods. One example of a differing strategy was presented by Zhang et al., who developed a novel approach to increase the adsorption kinetics and efficiency of their MIPs to the neonicotinoid paichongding (IPP) [68]. They aimed to increase the adsorption kinetics and capacity by coating the MIPs as a surface molecular imprinted film on a magnetic nanocore. Specifically, they used magnetic Fe_3_O_4_ nanoparticles coated with SiO_2_ and then vinyl-modified to form Fe_3_O_4_@SiO_2_@C=C magnetic nanoparticles. They coated their magnetic nanoparticles with a methacrylic acid polymer with ethylene glycol dimethacrylate crosslinkers in the presence of IPP to form their magnetic molecularly imprinted nanoparticles (MMIPs). Incorporation of the magnetic component was hypothesized to address the challenges of low binding capacity and slow mass transfer by having nanoparticles suspended in solution, increasing both the binding capacity and mass transfer rate, with subsequent application of an external magnetic field for MMIP separation. This combination of increased binding capacity and subsequent separation and concentration has applications in solid-phase extraction and also potentially in the development of chemical sensors. The researchers demonstrated a maximum adsorption capacity of 17.30 mg g^−1^ for their MMIP, with an increased mass transfer rate demonstrated by the decreased equilibrium time, with traditionally imprinted materials typically requiring 12–24 h and their MMIPs reaching equilibrium in 150 min. The researchers also found that their MMIPs lost only 2.25–6.55% of their adsorption capacity over four adsorption/desorption cycles, which demonstrated the reusability of the MMIPs. While the authors did not directly present a chemical sensor, their work presents a novel approach to increasing the binding capacity of MIPs to neonicotinoids, with potential applications in decreasing the response time and increasing the sensitivity of neonicotinoid sensors.

**Figure 7:**
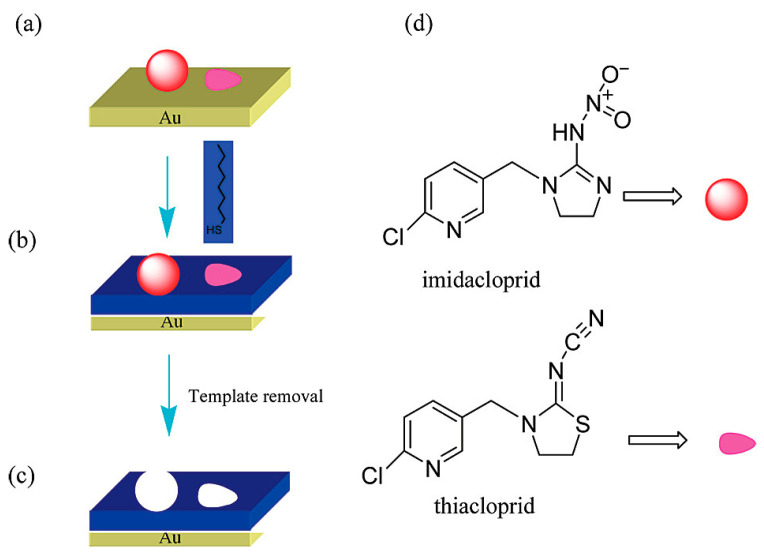
Formation of IMD and THC selective binding sites using 2D molecular imprinting on a gold surface. (**a**) Shows the preadsorption of IMD and THC to the gold surface, (**b**) shows the self-assembly of an alkanethiol around the templates, (**c**) shows the removal of both IMD and THC and the creation of two distinct binding sites, and (**d**) shows the molecular structures of both IMD and THC. Reproduced with permission from Bi et al. (2009) [69] Copyright © 2008 American Chemical Society.

Bi et al. also reported an IMD and THC sensor using a quartz crystal microbalance (QCM) [69]. They used 2D molecular imprinted monolayers with alkane thiols on a gold surface, synthesized with either IMD, THC, or both as template molecules. The template molecules were pre-adsorbed onto the gold surface, and self-assembly of alkanethiols around them resulted in the formation of two distinct binding pockets for IMD and THC, respectively. The overall synthesis process is illustrated in Figure 7.

**Figure 8:**
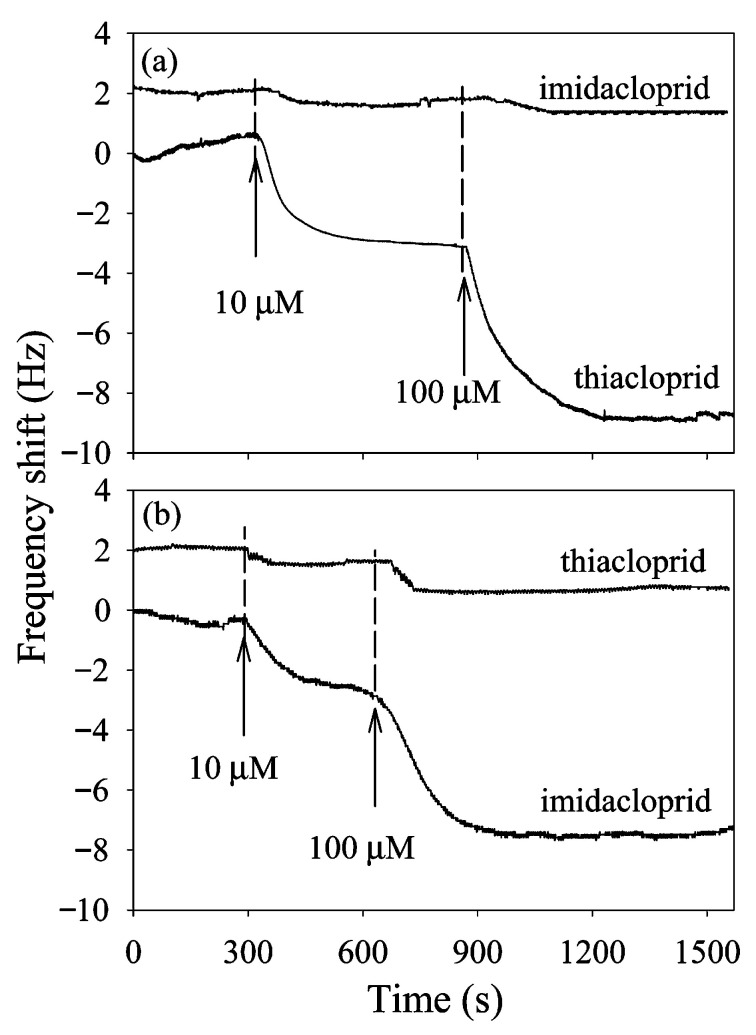
Detection of IMD and THC using a quartz crystal microbalance coated with a molecular imprinted monolayer. The top graph (**a**) shows the detection of IMD and THC using a THC-specific monolayer, and the bottom graph (**b**) shows similar detection using an IMD-specific monolayer. Reproduced with permission from Bi et al. (2009) [69] Copyright © 2008 American Chemical Society.

The mechanism of detection for QCM is the change in mass on the crystal, affecting the crystal’s resonant frequency. QCM is highly sensitive but unable to distinguish between various analytes. Thus, the authors combined a quartz crystal microbalance with a molecular imprinted monolayer for the selective recognition of IMD and THC. The authors were able to demonstrate real-time detection of IMD and THC in celery juice with an LOD of 10 µM, presenting a sensitive, selective, and fast response approach to neonicotinoid sensing in complex matrices. Detection of IMD and THC using a quartz crystal microbalance is shown in Figure 8.

## 5. Challenges and Limitations of MIPs

While MIPs have significant advantages applicable for sensor applications, such as good selectivity, strong physical robustness, low cost, good thermal stability, and easy preparation, there are major limitations associated with MIP technology that present challenges for the field of MIP-based neonicotinoid sensors. One of the major limitations of current MIPs is the heterogeneous distribution of binding sites, with varying affinities, specificities, and configurations. This heterogeneity of binding sites arises from the synthesis approach of integrating the template into the polymer matrix during polymerization. Heterogeneity of binding sites is most significant in MIPs that are synthesized using non-covalent template-monomer interactions, which are often favored in MIP synthesis due to their simplicity and fast template rebinding [70]. As a result, MIPs typically have an irregular distribution of binding sites within the polymer matrix, which has significant impacts on their binding affinities and specificities and thus on the sensitivity, selectivity, and performance of their respective sensors [71,72].

Modification of the MIP synthesis approach can partially address this issue of binding site heterogeneity; these synthesis approaches involve either covalent or semi-covalent template-monomer interactions. However, these approaches come with their own limitations. Covalent imprinting syntheses result in uniform distributions of binding sites within the polymer matrix, along with improved selectivity. However, the covalent template-monomer interactions result in the loss of many advantages associated with non-covalent interactions, as they are limited by slow template rebinding and more complicated synthetic procedures [70,73]. Semi-covalent interactions can address the issue of selectivity; however, many semi-covalent MIPs are susceptible to having incomplete cleavage of template molecules from the binding sites post-synthesis, which results in non-specific binding [74].

Another major challenge of using MIPs in sensors is the batch-to-batch reproducibility of both the binding sites and the immobilization of MIPs on sensor surfaces. The previously mentioned heterogeneity of binding sites results in significant batch-to-batch variability, as both the configuration and distribution of binding sites within the polymer matrix are inconsistent. This batch-to-batch variability arises not only from binding site heterogeneity but also from the process of immobilizing MIPs onto sensor surfaces, which further contributes to variability [71]. These challenges in batch-to-batch reproducibility from both processes can have significant impacts on the accuracy and reliability of any respective sensor [75].

Other issues that may arise include MIP rigidity and breakage during use or signal drift in the presence of complex matrices, which would provide inaccurate signals but could be mitigated through the use of additives [22,37]. Template leaching during use in the case of incomplete template removal is another significant issue, which could be reduced using thinner MIP sheets, to maintain consistent binding cavity distances [22]. Significant efforts have been made to address binding site heterogeneity and batch-to-batch reproducibility through improved imprinting precision. While various approaches exist, they often require substantial synthetic effort, including precise control over initiator, monomer, and cross-linkers, as well as temperature, stirring speed, solvent choice, and template proportion.

This limits the commercialization potential of MIP-based sensors, as one of their key advantages is rapid and efficient synthesis. However, emerging technologies such as computed tomography and ultrasonic scanning offer improved methods to structurally characterize the heterogeneity of binding sites. These advancements could help address imprinting precision challenges and further advance the field of MIP-based sensors [71,76].

## 6. Conclusions

Several neonicotinoid MIP-based sensing methods have been described, including electrochemical techniques such as voltammetry, potentiometry, photoelectrochemical, and impedance; optical techniques such as fluorescence and SERS; and less common techniques such as quartz crystal microbalance and magnetic solid-phase extraction. Although electrochemical MIP-based sensors were found to be the most robust with potential for miniaturization, all the highlighted methods show excellent potential in terms of their use of environmentally friendly materials, reusability, rapid detection, and low costs while maintaining high sensitivity and selectivity in the presence of interferents due to the use of MIPs. Particularly, recent advancements in optical MIP-based sensors have shown promising improvements in field-deployable detection through integration with smartphone-based imaging, paper-based strips, and magnetic separation systems. Innovations such as ratiometric fluorescence and dual-mode SERS/RRS sensors have significantly improved accuracy, signal robustness, and analyte specificity in complex matrices. These developments underscore the growing relevance of optical techniques in real-world neonicotinoid monitoring, complementing the analytical strength of electrochemical methods. Future work should be conducted to further improve these parameters, simplify sample preparation and analysis, explore the detection of less common neonicotinoids, and develop sensors for the simultaneous detection of multiple neonicotinoids. This could lead to significant advancements in sensor efficacy in the agricultural and food safety sectors.

## Figures and Tables

**Table 1:** Comparison of linear ranges and LODs for several neonicotinoids through various MIP-based electrochemical detection methods.

Electrode	Neonicotinoid	Detection Method	Linear Range (nM)	LOD (nM)	Ref.
Pyrrole MIP/IrO_2_/ITO	ACT	DPV	4.29–38.6	2.72	[16]
PoPD MIP/rGO/GCE	IMD	LSV	750–70,000	400	[15]
p-VBA MIP/GN/GCE	TMX	LSV	500–20,000	40	[21]
p-VBA MIP/GN/GCE	IMD	LSV	500–15,000	100	[22]
levodopa MIP/TiO_2_/GCE	IMD	SWV	2000–400,000	300	[23]
o-aminophenol/bromophenol blue MIP/Pt-In/GCE	IMD	DPV	0.20–50	0.012	[24]
4-vinylpyridine MIP/EGDMA/MWCNTs/carbon paste	ACT	DPV	0.001–5000	0.000033	[25]
PoPD MIP/Co/Mo_2_C/N-CNT/GCE	IMD	DPV	100–100,000	33	[26]
PoPD MIP/FcHT/AuNPs/GCE	IMD	DPV	500–100,000	47	[27]
PDA MIP/AgNPs/Fe_3_O_4_/GCE	ACT	DPV	450–8980	16	[28]
PoPD MIP/AQ/ZrO_2_-CNTs/GCE	IMD	DPV	50–10,000	14	[29]
Catechol MIP/poly(thionine)/β-CD/AMBC-3/GCE	DNF	DPV	50–10,000	16	[30]
PoPD MIP/poly(thionine)/MWCNTs/GCE	IMD	DPV	100–100,000	65	[31]
Chitosan/GDA MIP/AuNPs/rGO/Screen-printed carbon electrode	TMX IMD	DPV	500–3000	500	[33]
		DPV	1000–3000	500	[32]
PANI MIP/CNCs/CNT microneedle	IMD	CV DPV	2000–99,000	350	[34]
			200–92,000	60	
AM/EGDMA MIP/PVC ISE	DNF	Potentiometry	10^2^–10^7^	1.71	[35]
MAA/EGDMA MIP/rGO/GCE/PVC ISE	IMD	Potentiometry	5.0 × 10^2^–1.0 × 10^6^	200	[37]
PoPD MIP/WO_3_/CdS/FTO	CLT	PEC	1.0–5000	0.4	[40]
Chitosan/GDA MIP/ZnO/Bi_2_O_3_/Bi_2_S_3_/FTO	TMX	PEC	0.00070–0.70	0.000332	[41]
MAA/EGDMA MIP/poly(tyramine)/Au	IMD	Capacitance	5000–100,000	4610	[43]
PoPD MIP/WO_3_/MoS_2_/FTO	IMD	EIS	500–70,000	100	[45]

**Table 2:** Comparison of response times, selectivity, applications to real samples and stability for representative examples of neonicotinoid sensing through different MIP-based electrochemical detection methods.

Detection Method	Response Time	Selectivity (Interferents Tested)	Recovery (Real Samples)	Reproducibility (RSD (%)) *	Lifetime/Stability **	Ref.
DPV	8.60 s (coloring), 7.10 s (bleaching)	High (13 ions)	102.4–104.8 (tomato, salad, grapefruit, oranges)	0.44 (n = 10)	Maintained response after 100 cycles	[16]
LSV	10 min (including incubation)	High (4 neonicotinoids)	91.3–96.6 (pears)	4.5 (n = 7)	87% after 2 weeks	[15]
LSV	-	High (6 neonicotinoids, 6 ions)	75–78 (rice)	3.79 (n = 8)	95% after 2 weeks	[22]
DPV	-	High (4 neonicotinoids, 4 ions)	97.12–118.56 (tea)	4.69 (n = 5)	93% after 2 weeks	[26]
DPV	-	High (7 pesticides)	92.0–102 (tea, pear, lettuce, insecticidal spray)	2.5–2.9 (n = 10)	96.2% after 12 days	[30]
Potentiometry	<5 s	High (6 neonicotinoids, 4 ions)	96.5–106.4 (pesticides)	0.6 (n unavailable)	2 months	[37]
PEC	40 s	High (5 pesticides)	99.6–102.1 (water, soil)	1.56–3.74 (n = 5)	96.1% after 2 weeks	[41]
EIS	-	High (2 neonicotinoids, 2 ions)	93.3–109 (tap water)	4.2–7.6 (n = 3)	97.5% after 10 days	[45]

* Values *n* correspond to the number of replicates. ** Percentages correspond to the remaining signal intensities.

**Table 3:** Summary of optical detection methods for various MIP-based neonicotinoid sensors.

MIP Platform	Analyte	LOD (nM)	Linear Range (nM)	Response Time	Selectivity (Interferents Tested)	Recovery (Real Samples)	Reproducibility (RSD) *	Reusability	Stability	IF **	Ref.
ECL (UCNPs@ZIF-8)	IMD	3.9 × 10^−5^	3.9 × 10^−4^ –3900	8 min	High (6 pesticides/antibiotics)	95.0–105.2% (Fish, shrimp, lettuce)	1.0% (n = 7)	RSD 1.7% (7 days)	RSD 1.1% (12 cycles)	N/A	[51]
ECL (V-Ce-MOF nanowires on luminol/H_2_O_2_)	IMD	0.34	2–120	10 min	High (4 pesticides, 5 ions)	88.5–114.3% (Apples, bananas, tomatoes)	7.3% (n = 5)	N/A	<6.7% intensity change (120 min)	N/A	[55]
ECL (MIP/Tb-Ru-MOG/CeO_2_/N-GDY nanohybrid)	IMD	1.37	10–10,000	15 min	High (6 neonicotinoids)	83.9–102.8% (Tomato, broccoli, soil)	1.90% (n = 8)	73.7% retained (3 cycles)	88.4% retained (16 weeks)	N/A	[54]
Fluorescence (FMIHS strips with N-doped CDs/IOPCs)	IMD	0.25	0.39–196	20 min	High (4 pesticides and 6 IMD mixtures)	88.2–102.8% (Apples, cabbage, cucumber)	1.7% (n = 5)	RSD 1.3% (5 cycles)	RSD 1.2% (14 days)	5.9	[47]
Fluorescence (Fe_3_O_4_@SiO_2_@MIPIL core–shell)	IMD	0.3	1–1000	1 min	High (8 pesticides)	94.8–109.4% (Lake/canal water, apple, rice, cabbage)	N/A	Reusable (3 cycles)	N/A	N/A	[48]
Fluorescence (MIP@Si-CD nanocomposite)	ACT	2	7–107	2 min	High (6 analogs)	89.4–101.5% (Wastewater, apple)	2.7–3.3% (n = 5)	Reusable (5 cycles)	Stable (5 months)	9.8	[49]
Fluorescence (N-GQDs/PDA-MIP test strips)	THC	119	396–39,600	30 min	High (4 pesticides)	101–110% (Underground water)	N/A	N/A	N/A	N/A	[57]
Fluorescence (Eu (BTC)-MPS@MIP)	CLT	16	40–40,048	5 min	High (7 pesticides)	78.8–102.0% (Cabbage, vegetables, tomato, radish)	Good (n = 3)	85% signal (4 cycles)	Stable (6 weeks)	3.1	[58]
Fluorescence (UCNP@SiO_2_ core–shell MIP	ACT	37.3	89.8–3592	60 min	High (4 pesticides)	89.6–97.9% (Apple, strawberry)	N/A	N/A	Stable (6 days)	7.84	[59]
Fluorescence (CQD@MIPs and PD@MIPs)	ACT	CQD: 0.11 PD: 0.02	CQD: 0.36–64 PD: 0.08–109	11 min	High (11 pesticides + ACT mixtures)	92–103% (River/well/tap/seawater, apple)	N/A	N/A	N/A	N/A	[60]
Ratiometric (MIFP-SiCQDs@CdTe QDs)	IMD	13.9	19.6–1956	15 min	High (5 pesticides, 5 ions)	97.64–109.88% (River water, corn)	N/A	N/A	73% retained (17 days)	1.59	[46]
Ratiometric (B-CDs@MIPs/R-CDs)	TMX	13.5	50–25,000	30 min	High (5 pesticides, 4 ions)	91.4–105.7% (Fruit, cabbages, river water)	0.86% (n = 5)	N/A	Stable (3 months)	8.68	[61]
SERS (3D SDs/EMI/AgNP paper-based chip)	IMD	0.11	0.782–3129.8	2.5 min	High (5 pesticides)	89.0–104.1% (Chives, crown daisy, soybean, cucumber)	7.10% (n = 13)	RSD 6.22% (3 cycles)	94.6% retained (2 months)	N/A	[50]
SERS/RRS (MIP-HAuCl_4_-DA-HCl/AuNPs)	ACT	SERS: 0.0001	SERS: 0.075–0.75 RRS: 0.1–0.75	20 min	High (10 pesticides, 12 ions)	94.1–104% (Peach)	N/A	N/A	Stable (12 days)	N/A	[62]
SERS/RRS (Pd@MIP/AuNPs)	DNF	SERS: 0.03 RRS: 0.06	SERS: 0.00025–0.2 RRS: 0.0005–0.05	50 min	N/A	93–105% (Chili, orange, banana, etc.)	N/A	N/A	N/A	N/A	[63]
EC-SERS (MIP/AuNPs/ITO)	ACT	3.2	10–200,000	15 min	High (3 pesticides)	81.67–102.42% (Vegetables)	6.09% (n = 5)	N/A	80.55% retained (7 days)	N/A	[64]
SERS (Magnetic Fe_3_O_4_MIP/AuNPs)	ACT/THC	ACT: 151–309 THC: 94–144	ACT: 4490–8979 THC: 3957–7914	1 min	High (1 analog)	73.5–112.8% (Pear, peach)	N/A	Reusable (5–6 cycles)	N/A	N/A	[65]
SERS/RRS/Abs (Magnetic catalytic Fe_3_O_4_@MIP/AuNPs)	DNF	SERS: 0.009 RRS: 0.01 Abs: 0.35	SERS: 0.06–5.0 RRS: 0.06–5.0 Abs: 0.5–35	20 min	High (3 neonicotinoids, 3 ions)	98.0–111% (Rice, apples, tea)	N/A	N/A	RSD 6.7% (15 days)	N/A	[66]
SERS (MIP/AuNPs)	NIT	516.2–2733.2	3694–92,350	10 min	N/A	71.3–103.89% (Pear, peach, apple, tomato)	N/A	78.8% retained (5 cycles)	N/A	N/A	[67]

* Values *n* correspond to the number of replicates. ** N/A correspond to “not applicable” due to the absence of a reported IF value.

## Data Availability

No new data were created or analyzed in this study.

## Abbreviations

The following abbreviations are used in this manuscript:

ACT	Acetamiprid
AgNP	Ag nanoparticle
AM	Acrylamide
AMBC-3	Activated mung bean derived carbon
AQ	Anthraquinone
APTES	(3-aminopropyl)triethoxysilane
AuNP	Au nanoparticle
B-CD	Blue-emitting carbon dot
BTC	Benzene-1,3,5-tricarboxylate
CD	Carbon dot
CLT	Clothianidin
CNT	Carbon nanotube
CQD	Carbon quantum dot
CV	Cyclic voltammetry
DMIP	Dual-template molecularly imprinted polymer
DNF	Dinotefuran
DPV	Differential pulse voltammetry
ECL	Electrochemiluminescence
EGDMA	Ethylene glycol dimethyl acrylate
EIS	Electrochemical impedance spectroscopy
EMI	Electropolymerized molecular identifier
FcHT	6-(ferrocyanyl)hexanethiol
FMIHS	Fluorescence molecularly imprinted hydrogel strip
FTO	Fluorine-doped tin oxide
GCE	Glassy carbon electrode
GDA	Glutaraldehyde
GN	Graphene
HPLC	High-performance liquid chromatography
IOPC	Inverse opal photonic crystal
IMD	Imidacloprid
IPP	Paichongding
ISE	Ion-selective electrode
ITO	Indium tin oxide
LOD	Limit of detection
LSV	Linear sweep voltammetry
MAA	Methacrylic acid
MIFP	Molecularly imprinted fluorescent polymer
MIP	Molecularly imprinted polymer
MIT	Molecular imprinting technology
MMIP	Magnetic molecularly imprinted nanoparticles
MOF	Metal–organic framework
MOG	Metal–organic-gel
MWCNT	Multi-walled carbon nanotube
N-GQD	Nitrogen-doped graphene quantum dot
NIT	Nitenpyram
p-VBA	p-vinylbenzoic acid
PDA	Polydopamine
PD	Polymer dot
PEC	Photoelectrochemical
PL	Photoluminescence
PoPD	Poly(o-phenylenediamine)
PVC	Polyvinyl chloride
QD	Quantum dot
R-CD	Red-emitting carbon dot
RRS	Resonance Rayleigh scattering
rGO	Reduced graphene oxide
SEM	Scanning electron microscope
Si-CD	Silane-doped carbon dot
SiCQD	Silicon-carbon quantum dot
SPR	Surface plasmon resonance
SWV	Square wave voltammetry
THC	Thiacloprid
TMOPMAS	3-(trimethoxysilyl)propyl methacrylate
TMX	Thiamethoxam
UCNP	Upconversion nanoparticle
UCNP@SiO_2_	Silica-coated upconversion nanoparticle
UMV	Ultrafine mixed-valence
ZIF-8	Zeolitic imidazolate framework-8
β-CD	β-cyclodextrin
